# A 25-Year Retrospective of the Use of AI for Diagnosing Acute Stroke: Systematic Review

**DOI:** 10.2196/59711

**Published:** 2024-09-10

**Authors:** Zhaoxin Wang, Wenwen Yang, Zhengyu Li, Ze Rong, Xing Wang, Jincong Han, Lei Ma

**Affiliations:** 1 Nantong University Nantong China

**Keywords:** acute stroke, artificial intelligence, AI, machine learning, deep learning, stroke lesion segmentation and classification, stroke prediction, stroke prognosis

## Abstract

**Background:**

Stroke is a leading cause of death and disability worldwide. Rapid and accurate diagnosis is crucial for minimizing brain damage and optimizing treatment plans.

**Objective:**

This review aims to summarize the methods of artificial intelligence (AI)–assisted stroke diagnosis over the past 25 years, providing an overview of performance metrics and algorithm development trends. It also delves into existing issues and future prospects, intending to offer a comprehensive reference for clinical practice.

**Methods:**

A total of 50 representative articles published between 1999 and 2024 on using AI technology for stroke prevention and diagnosis were systematically selected and analyzed in detail.

**Results:**

AI-assisted stroke diagnosis has made significant advances in stroke lesion segmentation and classification, stroke risk prediction, and stroke prognosis. Before 2012, research mainly focused on segmentation using traditional thresholding and heuristic techniques. From 2012 to 2016, the focus shifted to machine learning (ML)–based approaches. After 2016, the emphasis moved to deep learning (DL), which brought significant improvements in accuracy. In stroke lesion segmentation and classification as well as stroke risk prediction, DL has shown superiority over ML. In stroke prognosis, both DL and ML have shown good performance.

**Conclusions:**

Over the past 25 years, AI technology has shown promising performance in stroke diagnosis.

## Introduction

### Background

Stroke is a global public health issue, ranking as the second leading cause of death and the third leading cause of disability and death. One in every 4 people aged >25 years will experience a stroke in their lifetime. Stroke is responsible for 11.6% of deaths, and its incidence, mortality, and disability rates are on the rise [1-3].

Acute stroke refers to the clinical pathological state caused by the acute disruption of cerebral blood vessels. It can result in either the interruption of blood supply to the brain or the rupture of brain vessels, leading to damage to brain tissue. Ischemic strokes account for approximately 80% of all strokes, while hemorrhagic strokes make up approximately 20% [4]. Ischemic stroke is caused by reduced blood flow or blockage in the cerebral vessels, leading to oxygen and blood deprivation in brain tissue. Hemorrhagic stroke results from bleeding due to the rupture of cerebral vessels, with symptoms typically appearing within minutes and potentially leading to severe neurological deficits [5].

**Figure 1:**
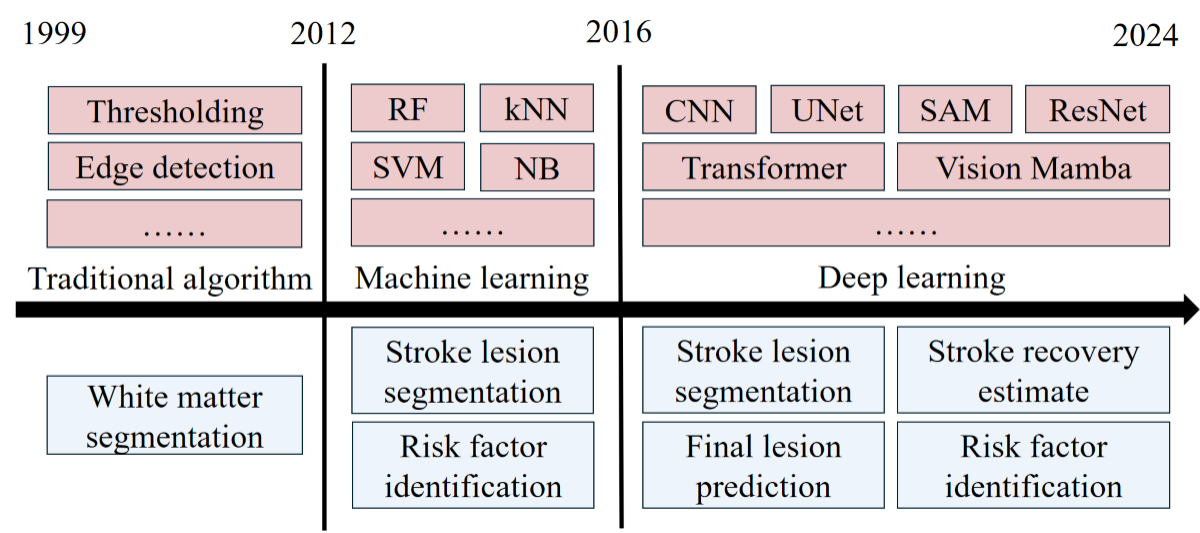
Artificial intelligence used for acute stroke diagnosis over the past 25 years. CNN: convolutional neural network; kNN: k-nearest neighbors; NB: naive Bayes; ResNet: residual network; RF: random forest; SAM: Segment Anything Model; SVM: support vector machine.

Over the past 25 years, artificial intelligence (AI) technology has achieved remarkable progress across various domains, notably in medical diagnostics [6]. Early AI applications primarily used rule-based systems and machine learning (ML) models to analyze medical data and predict outcomes. With the advent of deep learning (DL), AI’s ability to handle complex medical imaging data improved significantly. DL models, such as convolutional neural networks (CNNs), have been particularly effective in automatically identifying stroke lesions and detecting stroke risk factors in medical images. These models use large data sets to learn intricate patterns and features, improving diagnostic accuracy and efficiency. In addition, by integrating vast amounts of clinical data with imaging data, AI can now predict patient prognosis, assess functional recovery, and forecast treatment outcomes with greater precision [7] (Figure 1).

The swift and precise analysis of imaging data by AI offers detailed insights into the patient’s condition, significantly supporting physicians in diagnostic decision-making and treatment planning. These AI models promote a deeper understanding of disease progression, enabling the development of personalized rehabilitation plans and thereby improving long-term patient outcomes [8].

**Figure 2:**
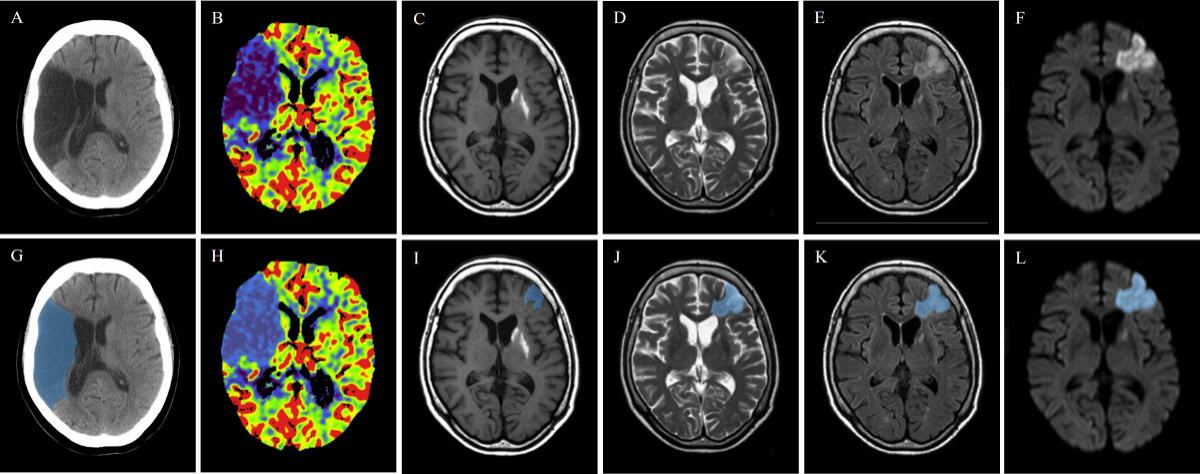
Stroke lesions identified using different imaging modalities. (A) Noncontrast computed tomography (NCCT). (B) CT perfusion (CTP). (C) T1-weighted imaging (T1WI). (D) T2-weighted imaging (T2WI). (E) Fluid-attenuated inversion recovery (FLAIR). (F) Diffusion-weighted imaging (DWI). (G) NCCT (annotated). (H) CTP (annotated). (I) T1WI (annotated). (J) T2WI (annotated). (K) FLAIR (annotated). (L) DWI (annotated). Stroke lesion areas are marked in blue, and the images are not paired.

Computed tomography (CT) and magnetic resonance imaging (MRI) are the most commonly recommended imaging methods for the clinical diagnosis of acute stroke. MRI has higher clarity and clinical sensitivity, offering better soft tissue contrast [9]. MRI imaging techniques used for brain examinations include T1-weighted imaging (T1WI), T2-weighted imaging (T2WI), fluid-attenuated inversion recovery (FLAIR), and diffusion-weighted imaging (DWI). T1WI provides excellent anatomical detail, but it is not very sensitive to early changes in acute stroke. T2WI can highlight increased water content in brain tissue and is commonly used for evaluating the subacute and chronic stages of stroke, although the images may be blurry and have artifacts. FLAIR can suppress cerebrospinal fluid signals, making it suitable for detecting white matter lesions and identifying lesions near the cerebrospinal fluid pathways. However, it has longer scanning times and is not sensitive enough for detecting small infarcts. DWI is highly sensitive to early acute cerebral ischemia, but DWI images have lower resolution and are not sufficiently sensitive to small infarcts [10]. Compared to MRI imaging, CT is faster and commonly used for detecting early signs of infarction. CT angiography (CTA) can provide information about vascular occlusion to guide treatment decisions, while CT perfusion (CTP) imaging can assess the extent of ischemic core and penumbra areas [11]. The 2018 American Heart Association and American Stroke Association guidelines indicate that noncontrast CT (NCCT) and CTA are recommended within 6 hours of acute stroke onset, while MRI and CTP are recommended for the 6- to 24-hour window [12,13] (Figure 2).

**Figure 3:**
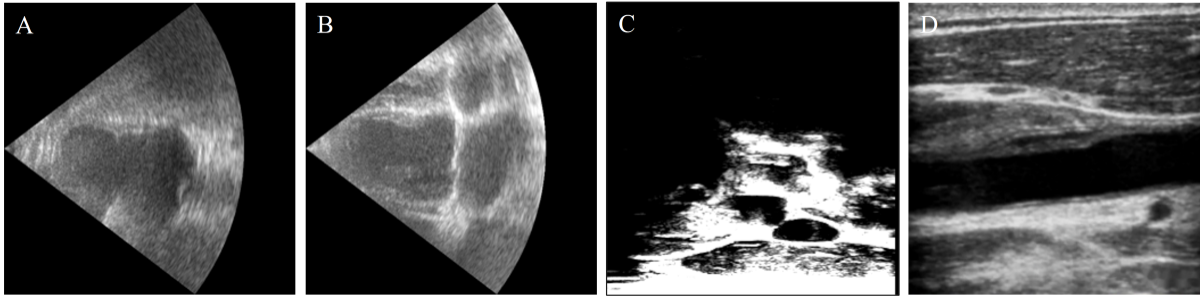
Cardiac ultrasound (US) and carotid US. (A) Cardiac US (2 heart chambers). (B) Cardiac US (4 heart chambers). (C) 3D carotid US. (D) 2D carotid US.

Ultrasound examination, with its advantages of fast imaging speed, lack of radiation, and lower cost, is commonly used in clinical practice for cardiac and carotid artery assessments [14,15] (Figure 3). For cerebral small vessel evaluation, T2WI and FLAIR-weighted MRI are commonly used in clinical settings [16].

### Main Contributions of This Review

The main contributions of this review are as follows: first, it provides a comprehensive summary of the common applications of AI in the diagnosis of acute stroke. Second, it outlines the development trends of AI algorithms for stroke lesion segmentation and classification, stroke risk prediction, and stroke prognosis from 1999 to 2024. This includes an analysis of data sources, types of algorithms, outcome metrics, and qualitative results described in each included article. Third, it discusses the clinical significance of the findings, existing challenges, and future research directions in this rapidly advancing field. The review aims to provide researchers and clinicians with insights into the current state of acute stroke diagnosis based on DL, offering a comprehensive reference for clinical practice.

## Methods

### Overview

For this systematic review, we adhered to the PRISMA (Preferred Reporting Items for Systematic Reviews and Meta-Analyses) 2020 guidelines [17]. We conducted a comprehensive literature search from January 1999 to February 2024 across the Google Scholar, PubMed, IEEE Xplore, Web of Science, and SpringerLink databases. The Boolean search string used was as follows: (ABSTRACT (“artificial intelligence” OR “AI” OR “machine learning” OR “deep learning” OR “CNN”) AND ABSTRACT (“ischemic stroke” OR “hemorrhagic stroke” OR “acute stroke” OR “stroke”) AND ABSTRACT (“magnetic resonance imaging” OR “MRI” OR “Computed Tomography” OR “CT” OR “Ultrasound” OR “US”)).

### Evaluation Metrics

To assist readers in gaining a clearer understanding of these articles, we will define the model evaluation metrics featured in this review (Textbox 1). Common metrics include the Dice similarity coefficient (DSC) [18], Hausdorff distance [19], area under the curve (AUC) [20], accuracy [21], positive predictive value [22], recall [22], and specificity [22]. For image segmentation tasks, the main focus is on the DSC and Hausdorff distance metrics. For image classification tasks and prediction tasks, the main focus is on the accuracy, positive predictive value, recall, specificity, and AUC metrics (Table 1).

In this review, we also examine the statistical measures of significance and uncertainty, robustness or sensitivity analysis, methods for explainability or interpretability, and their validation (including validation or testing on external data), if reported in the studies [23].

**Acronyms with their definitions.**

Acronyms and definitions

- AF: atrial fibrillation
- AI: artificial intelligence
- ASPECTS: Alberta Stroke Program Early CT Score
- AUC: area under the curve
- CAS: carotid artery stenosis
- CE: cardioembolism
- CNN: convolutional neural network
- CSVD: cerebral small vessel disease
- CT: computed tomography
- CTA: computed tomography angiography
- CTP: computed tomography perfusion
- DL: deep learning
- DNN: deep neural network
- DSC: Dice similarity coefficient
- DT: decision tree
- DWI: diffusion-weighted imaging
- FCN: fully convolutional network
- FLAIR: fluid-attenuated inversion recovery
- GRU: gated recurrent unit
- HD: Hausdorff distance
- kNN: k-nearest neighbors
- LA: left atrium
- LAA: large artery atherosclerosis
- LLM: large language model
- MHCA: multihead cross-attention
- ML: machine learning
- MRF: Markov random field
- MRI: magnetic resonance imaging
- NB: naive Bayes
- NCCT: noncontrast computed tomography
- PCA: principal component analysis
- PPV: positive predictive value
- RBF: radial basis function
- RBM: restricted Boltzmann machine
- RF: random forest
- ROI: region of interest
- SAM: Segment Anything Model
- SAO: small artery occlusion
- SE: squeeze and excitation
- STRIVE-1: Standards for Reporting Vascular Changes on Neuroimaging-1
- SVM: support vector machine
- T1WI: T1-weighted imaging
- T2WI: T2-weighted imaging
- US: ultrasound
- VIT: vision transformer
- VMamba: Vision Mamba
- WMH: white matter hyperintensities

**Table 1:** Equations and significance for evaluation metrics.

Metric		Equation	Significance
**Segmentation metrics**			
	DSC^a^ [18]	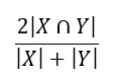	The overlap between segmentation results and labels; X and Y represent the segmentation result and label, respectively
	HD^b^ [19]	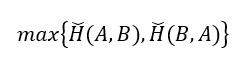	The maximum boundary distance between results and labels; 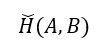 denotes the directed Hausdorff distance from set A to set B
**Classification and prediction metrics**			
	AUC^c^ [20]	—^d^	Threshold-independent classification performance
	Accuracy [21]	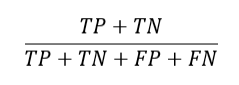	The proportion of correct predictions among all cases; TP represents true positive, TN represents true negative, FP represents false positive, and FN represents false negative
	PPV^e^ [22]	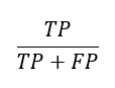	The proportion of TPs among predicted positives
	Recall^f^ [22]	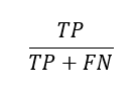	The proportion of TPs among all actual positives
	Specificity [22]	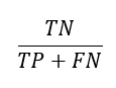	The proportion of TNs among all actual negatives

^a^DSC: Dice similarity coefficient.

^b^HD: Hausdorff distance.

^c^AUC: area under the curve.

^d^Not applicable.

^e^PPV: positive predictive value (can also be referred to as *precision*).

^f^Recall can also be referred to as *sensitivity*.

## Results

### Literature Search Results

#### Overview

**Figure 4:**
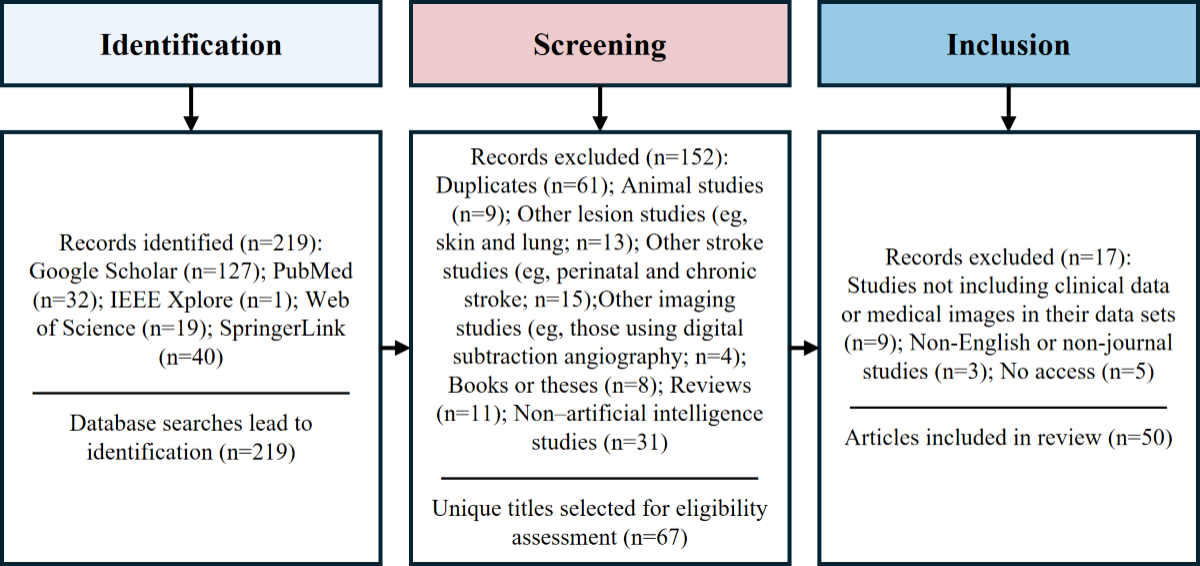
PRISMA (Preferred Reporting Items for Systematic Reviews and Meta-Analyses) flowchart for systematic filtering and selection of articles.

In the initial identification phase of the PRISMA flowchart (Figure 4), we identified 219 articles. During the screening phase, we reviewed the titles and abstracts of these publications to remove duplicates. We also excluded articles involving animals, other lesion types (eg, skin and lung), other stroke types (eg, perinatal stroke and chronic stroke), and other imaging modalities (eg, digital subtraction angiography). In addition, books, theses, review articles, and studies that did not propose an automated method based on ML or DL were excluded.

In the final inclusion phase, of the initial 219 articles, we retained 50 (22.8%) unique articles. These articles were thoroughly read to assess their eligibility for inclusion in this review. Articles were selected if they (1) relied on ML-based or DL-based automated methods and (2) included clinical records or medical images.

#### Segmentation and Classification of Stroke Lesions

##### Overview

Imaging examinations are typically included in the admission tests for patients with acute stroke [24,25]. Segmentation of stroke lesions can determine lesion volume and lesion location [26]. Classification of stroke lesions allows for the rapid identification of stroke type, aiding in patient triage [27]. This supports disease diagnosis, assists physicians in formulating treatment plans based on the patient’s clinical presentation, and provides guidance for surgical or pharmacological interventions (Table 2).

**Table 2:** Methods and performance of stroke lesion segmentation and classification.

Study, year		Image type	Data set, n	Methods	Performance	Merits
**Machine learning (MRI^a^)**						
	Maier et al [28], 2014	Multimodal MRI	36	SVM^b^	DSC^c^: 0.74	Uses SVM to extract local features
	Mitra et al [29], 2014	Multimodal MRI	36	B-MRF^d^, RF^e^	DSC: mean 0.39 (SD 0.22)	Multistage algorithm; fuses T1WI^f^, T2WI^g^, FLAIR^h^, and DWI^i^
	Maier et al [30], 2015	Multimodal MRI	37	RF	DSC: 0.65; HD^j^: 28.61 mm	Uses extra trees for voxel-level classification; incorporates intensity-derived image features
	Pustina et al [31], 2016	T1WI	105	RF	DSC: mean 0.696, SD 0.16; HD: mean 17.9, SD 9.8 mm	Combines RF with multiresolution neighborhood data analysis
	Griffis et al [32], 2016	T1WI	30	GNB^k^	DSC: 0.66	Incorporates probabilistic tissue segmentation; incorporates image algebra
**Deep learning (CT^l^)**						
	Goncharov et al [33], 2018	CTP^m^	156	UNet	DSC: mean 0.53, SD 0.01; HD: mean 23.29, SD 0.02 mm	Integrates UNet and T-Net (a variation of the UNet architecture); incorporates residual blocks
	Clèrigues et al [34], 2019	CTP	156	CNN^n^	DSC: mean 0.49, SD 0.31; HD: mean 11.3, SD 31.6 mm	Uses symmetric modality augmentation; applies uncertainty filtering
	Wang et al [35], 2020	Multimodal CT	156	CNN	DSC: mean 0.51, SD 0.31; HD: mean 0.55, SD 0.34 mm	Uses an innovative loss function; extracts features from CTA^o^ images, synthesized pseudo-DWI
	Kuang et al [36], 2021	NCCT^p^	230	CNN	DSC: 0.448	Combines CNN and classification network; introduces the ASPECTS^q^ scale
	Soltanpour et al [37], 2021	CTP	156	UNet	DSC: 0.69	Uses multiscale convolutional blocks, incorporates contralateral CT images; uses time-to-maximum heat maps
	Luo et al [38], 2021	NCCT	293	Transformer, ResNet^r^-50	DSC: 0.7358	Combines ResNet-50 and transformer; introduces multihead cross-attention module
	De Vries et al [39], 2023	CTP	156	UNet	DSC: 0.564	Incorporates symmetry-aware spatiotemporal convolutional structure; uses the dynamics of cerebral microperfusion; incorporates attention in skip connections
	Kuang et al [40], 2024	NCCT	482	Transformer, CNN	DSC 0.5407	Introduces hybrid CNN transformer; introduces bilateral difference learning
**Deep learning (MRI)**						
	Chen et al [41], 2017	DWI	741	CNN	DSC: 0.67	Multistage algorithm; introduces multiscale concept
	Zhang et al [42], 2018	DWI	242	DenseNet	DSC: 0.7913	Combines dense connections and multiscale context
	Karthik et al [43], 2019	Multimodal MRI	28	FCN^s^	DSC: 0.70	Uses leaky rectified linear unit in last 2 layers
	Clèrigues et al [44], 2020	Multimodal MRI	236	UNet	DSC: 0.59, SD 0.31; HD: 0.84, SD 0.10 mm	Uses symmetric modality enhancement; uses dynamic weighted loss functions
	Wu et al [45], 2023	Multimodal MRI	490	Transformer	DSC: 0.7258	Uses point-wise rendering to compute boundary
	Wu et al [46], 2023	Multimodal MRI	490	Transformer, CNN	DSC: 0.7368	Multistage algorithm; proposes 2 edge enhancement modules
	Soh et al [47], 2023	T1WI	239	Transformer, UNet	DSC: 0.737	Comprises 2 parallel pipelines
**Stroke classification**						
	Adam et al [48], 2016	NCCT	400	DT^t^, CNN	AUC^u^: 0.994; recall: 0.987	Compares kNN^v^ and DT
	Gautam and Raman [49], 2021	NCCT	900	CNN	ACC: 98.33	Uses multifocus image fusion preprocessing
	Chen et al [50], 2022	NCCT	400	CNN	Recall: 0.99	Uses hyperparameter optimization; uses transfer learning; compares optimized CNN and ResNet-50

^a^MRI: magnetic resonance imaging.

^b^SVM: support vector machine.

^c^DSC: Dice similarity coefficient.

^d^B-MRF: Bayesian-Markov random field.

^e^RF: random forest.

^f^T1WI: T1-weighted imaging.

^g^T2WI: T2-weighted imaging.

^h^FLAIR: fluid-attenuated inversion recovery.

^i^DWI: diffusion-weighted imaging.

^j^HD: Hausdorff distance.

^k^GNB: Gaussian naive Bayes.

^l^CT: computed tomography.

^m^CTP: computed tomography perfusion.

^n^CNN: convolutional neural network.

^o^CTA: computed tomography angiography.

^p^NCCT: noncontrast computed tomography.

^q^ASPECTS: Alberta Stroke Program Early Computed Tomography Score.

^r^ResNet: residual network.

^s^FCN: fully convolutional network.

^t^DT: decision tree.

^u^AUC: area under the curve.

^v^kNN: k-nearest neighbors.

#### Stroke Lesion Segmentation Based on ML

Stroke lesion segmentation is a challenging task due to the blurred boundaries between stroke lesions and normal tissues, particularly in hemorrhagic strokes. MRI images offer greater contrast and clarity compared to CT scans, allowing for more clearly delineated observation of stroke lesion boundaries [51].

Traditional thresholding and heuristic algorithms are inadequate for accurately segmenting stroke lesions. Before 2012, there was limited research on stroke lesion segmentation [52,53].

From 2012 to 2016, stroke lesion segmentation algorithms were primarily based on ML algorithms, with common segmentation methods mainly involving support vector machines (SVMs) [54], random forests (RFs) [55], and naive Bayes [56]. Due to the limited feature extraction capabilities of ML segmentation algorithms during this period, these algorithms primarily used MRI images.

In 2014, Maier et al [28] proposed an image segmentation method for ischemic stroke lesions based on SVMs using multimodal MRI images. The authors used SVMs to extract local features from the multimodal MRI data [28]. In the same year, Mitra et al [29] used a Bayesian-Markov random field for preliminary FLAIR image classification, then analyzed multimodal MRI data (T1WI, T2WI, FLAIR, and DWI) and contextually relevant features using an RF to identify likely lesion areas.

One year later, Maier et al [30] proposed another method for segmenting ischemic stroke lesions in multimodal MRI images using extra tree forests. This approach involved using extra trees for voxel-level classification and incorporated intensity-derived image features [30].

In 2016, Pustina et al [31] proposed an automatic segmentation algorithm for stroke lesions in T1WI images, combining RFs with multiresolution neighborhood data analysis to enhance efficiency and reduce observer dependency. In the same year, Griffis et al [32] used a stroke lesion segmentation method based on the Gaussian naive Bayes classifier. By using probabilistic tissue segmentation and image algebra to create feature maps, the authors encoded information about missing and abnormal tissues and used the leave-one-out method for cross-validation [32].

#### Stroke Lesion Segmentation Based on DL

After 2016, there was a leap in the capabilities of DL for medical segmentation and feature extraction. DL has since outperformed ML as the main force in stroke image segmentation [57]. Due to the improved feature extraction capabilities of DL, algorithms for stroke lesion segmentation based on CT scans have emerged.

In 2018, Goncharov et al [33] proposed a CNN framework integrating UNet and T-Net (a variation of the UNet architecture). In each network, convolutional layers were replaced with residual blocks, and an initial convolution layer was added to enhance the learnability of the deep network [33].

In 2019, Clèrigues et al [34] proposed a 3D CTP segmentation method based on a 2D asymmetrical residual CNN. This method addresses issues such as small sample size, class imbalance, and overfitting by enhancing training, using symmetric modality augmentation, and applying uncertainty filtering, thereby improving performance and achieving fast inference [34].

In 2020, Wang et al [35] proposed a multimodal 3D CTP segmentation method based on CNNs. The authors extracted features from CTA images, synthesized pseudo-DWI, and used an innovative loss function to focus on lesion areas, thereby improving segmentation accuracy [35].

In 2021, Kuang et al [36] proposed a DL approach named EIS-Net, which combines a 3D triple CNN and a multiregion classification network for automatic segmentation of early ischemic stroke and Alberta Stroke Program Early CT Score scoring on NCCT in patients with acute ischemic stroke. In the same year, Soltanpour et al [37] proposed an improved version of the UNet network called MultiResUNet for the automatic segmentation of ischemic stroke lesions in CTP images. The algorithm uses multiscale convolutional blocks and incorporates contralateral CT images as references, estimating lesion locations using time-to-maximum heat maps [37]. Luo et al [38] proposed an ischemic stroke lesion segmentation method called UCATR for NCCT images. This method combines residual network (ResNet)-50 (a CNN that is 50 layers deep) and transformer encoders and introduces a multihead cross-attention module in the decoder to improve the accuracy of spatial information recovery [38].

In 2023, de Vries et al [39] proposed a model called Perf-UNet for segmenting the infarct core area from CTP source data. The model uses a symmetry-aware spatiotemporal convolutional structure, leveraging the dynamics of cerebral microperfusion, and incorporates attention modules in the skip connections [39].

In 2024, Kuang et al [40] proposed a hybrid CNN-transformer network based on circular feature interactions and bilateral difference learning. This network introduces a hybrid CNN-transformer in the encoder, a recurrent feature interaction module, and a shared CNN decoder with bilateral difference learning modules [40].

Compared to CT-based stroke lesion segmentation, MRI-based stroke lesion segmentation achieves higher segmentation accuracy.

In 2017, Chen et al [41] proposed a CNN network composed of a deconvolutional network for initial segmentation and a multiscale convolutional label evaluation network for eliminating false positives in small lesions.

In 2018, Zhang et al [42] proposed FC-DenseNet for segmenting acute stroke lesions in DWI medical images. This model combines dense connections and multiscale context on top of a 3D CNN to address common issues in DWI, such as noise, artifacts, and variations in lesion size and location [42].

In 2019, Karthik et al [43] proposed an improved fully convolutional network that applies leaky rectified linear unit activation in the last 2 layers of the UNet architecture. This approach precisely reconstructs ischemic lesions and allows the network to learn additional features not considered in the original UNet architecture [43].

In 2020, Clèrigues et al [44] addressed class imbalance issues by using symmetric modality enhancement, balanced training sample strategies, and dynamic weighted loss functions in the UNet architecture.

In 2023, Wu et al [45] introduced a lesion boundary–rendering method named TransRender. This method uses transformers to capture global information during the encoding phase and uses multiple renderings to effectively map encoding features of different levels to the original spatial resolution. The method adaptively selects points to compute boundary features based on point-wise rendering, supervising the rendering module to generate points and continuously refine uncertain regions [45]. In the same year, Wu et al [46] proposed a 2-phase brain multimodal MRI lesion segmentation method named W-Net. This method uses a CNN and a transformer as backbone networks, introduces a boundary deformation module and a boundary constraint module to address boundary ambiguity, and designs a multitask learning loss function to optimize W-Net from both regional and boundary perspectives [46]. Soh et al [47] proposed an algorithm named hybrid UNet transformer. This algorithm comprises 2 parallel pipelines, where the transformer-based pipeline uses feature maps from the intermediate layers of the UNet-based pipeline to enhance feature extraction capabilities [47].

#### Stroke Classification

The admission evaluation for patients with acute stroke typically includes stroke classification. Patients with acute ischemic stroke who are eligible for thrombolytic therapy should be referred to a thrombolytic center for treatment. For patients who are not suitable for thrombolytic therapy, conservative treatment should be provided upon admission. Patients with hemorrhagic stroke require rapid intervention to minimize brain damage and improve survival chances [24].

**Figure 5:**
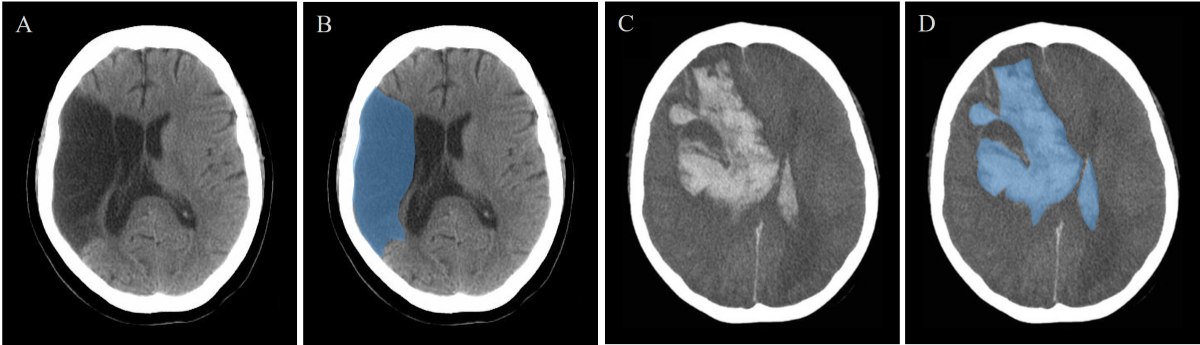
Ischemic stroke and hemorrhagic stroke. (A) Ischemic stroke. (B) Ischemic stroke (annotated). (C) Hemorrhagic stroke. (D) Hemorrhagic stroke (annotated). Stroke lesion areas are marked in blue.

Acute ischemic stroke classification is typically based on CT imaging [58]. In CT images, ischemic stroke lesions appear as low-density regions (dark areas), while hemorrhagic stroke lesions appear as high-density regions (white areas; Figure 5) [48-50].

Adam et al [48] proposed a stroke lesion classification model based on decision tree and k-nearest neighbors (kNN) algorithms. The study found that the decision tree algorithm outperformed kNN in classification [48].

Gautam and Raman [49] proposed a CNN model for classifying stroke CT images. The authors improved the quality of CT images by using multifocus image fusion preprocessing and then inputted the processed images into a 13-layer CNN architecture for classification [49].

Chen et al [50] used hyperparameter optimization and transfer learning to identify stroke conditions in brain CT images. The optimized CNN and ResNet-50 models demonstrated high accuracy in distinguishing between normal, hemorrhagic stroke, ischemic stroke, and other lesions. Although ResNet-50 exhibited the highest accuracy, it required more processing time [50].

#### In-Depth Analysis of Stroke Lesion Segmentation and Classification

In clinical practice, the choice between using MRI and CT depends on the specific condition of the patient. CT has lower sensitivity in identifying early changes of acute ischemic stroke but demonstrates higher sensitivity in detecting characteristic high-density lesions of hemorrhagic stroke [59]. Compared to CT, MRI has higher contrast and a stronger ability to differentiate between brain tissues. The DWI sequence can detect minor changes in brain tissue just minutes after ischemia occurs. MRI also provides clearer identification of small hemorrhages and better differentiation between hemorrhagic and ischemic areas, along with detailed information on the extent and type of brain tissue damage after a stroke [60].

Before 2012, brain segmentation algorithms were primarily based on traditional methods for brain white matter segmentation. There are few studies focusing on the segmentation of stroke lesions in this period [52,53]. Between 2012 and 2016, segmentation algorithms for ischemic stroke lesions were primarily based on ML. Due to the blurred boundaries between stroke lesions and surrounding normal tissue in CT images, algorithms from this period mainly focused on MRI imaging. The accuracy of segmentation algorithms during this time generally did not exceed 0.75. These algorithms were complex, had weak generalization capabilities, and could not achieve the level of accuracy of manual segmentation. After 2016, with the development of UNet, DL has shown superior performance in the segmentation of stroke lesions [57]. As DL enables more in-depth feature extraction from medical images, significantly enhancing the extraction of effective features, an increasing number of researchers have started focusing on the segmentation of ischemic stroke lesions in CT images. However, the accuracy of these segmentation algorithms based on CT commonly did not surpass 0.75. Algorithms for segmenting ischemic stroke lesions based on MRI have shown better accuracy, enough to be comparable to manual segmentation. Some algorithms have achieved accuracies of up to 0.85. To facilitate the triage of patients with acute stroke, due to the urgency of the condition, the classification of stroke types primarily relies on CT images [58]. Current algorithms have achieved high accuracies, generally exceeding 0.90, indicating promising application prospects.

Data preprocessing is important for medical image segmentation. Preprocessing aims to standardize image quality, enhance valuable features, and reduce segmentation difficulties. Clèrigues et al [34] and Clèrigues et al [44] used symmetry-enhanced modalities, leveraging the high bilateral symmetry exhibited by the human brain in its natural state. This approach provides clearer and more accurate information for subsequent steps such as feature extraction, lesion detection, and image segmentation. For complex segmentation tasks such as stroke lesion segmentation, the introduction of multimodal approaches can significantly enhance algorithm performance by extracting complementary information from different imaging techniques. The studies by Mitra et al [29] and Wang et al [35] are particularly representative. Mitra et al [29] used multimodal MRI data, including T1WI, T2WI, FLAIR, and DWI, along with context-aware features. Wang et al [35] extracted features from CTA images, synthesized pseudo-DWI, for use in CTP segmentation methods.

In addition, some studies used multistage algorithms, combining coarse and fine-grained approaches to achieve better segmentation performance; for instance, Mitra et al [29] used a Bayesian-Markov random field model for preliminary classification of FLAIR images and used RFs for multimodal MRI segmentation. Chen et al [41] used a deconvolutional network for initial segmentation and used a multiscale convolutional label evaluation network to evaluate and eliminate false positives of small lesions, achieving promising results on large data sets and demonstrating the algorithm’s generalization ability.

Since 2021, various attention mechanisms and transformers have been widely applied in the field of medical image segmentation, leading to further improvements in the performance of stroke lesion segmentation algorithms. Transformers leverage self-attention mechanisms, enabling neural networks to capture global information and exhibit excellent performance when processing long sequence data [61]. De Vries et al [39] introduced attention mechanisms on the skip connections between the encoder and decoder, reducing the time dimension and only propagating the most informative features. Luo et al [38], Kuang et al [40], Wu et al [45], Wu et al [46], and Soh et al [47] all incorporated the concept of transformers, leading to significant improvements in algorithm performance.

### Stroke Risk Prediction

#### Overview

Clinical methods for stroke risk prediction include clinical risk scores (eg, Framingham Stroke Risk Profile [62]), genetic risk analysis [63], serum biomarker detection [64], and imaging analysis [65]. Compared to other methods, imaging analysis provides direct anatomical and functional information, allowing clinicians to observe pathological conditions directly. In addition, imaging data can quantify stroke risk factors such as atrial fibrillation (AF), carotid artery stenosis (CAS), and cerebral small vessel disease, which are crucial for stroke risk prediction [66]. Furthermore, imaging techniques can monitor disease progression and treatment effects in real time, enabling clinicians to adjust treatment strategies promptly. Due to these advantages of imaging techniques, AI-based stroke risk prediction using medical imaging has become a major focus of research (Table 3).

**Table 3:** Methods and performance of stroke prediction.

Study, year		Image type	Data set, n	Methods	Performance	Merits
**AI ^a^-assisted detection of CAS^b^**						
	Alam et al [67], 2015	US^c^	200	RBF^d^	Accuracy: 0.982	Combines fuzzy c-means clustering; incorporates noise-handling methods
	Araki et al [68], 2017	US	407	SVM^e^	Accuracy: 0.9428	Uses spatial domain filtering
	Al-Mohannadi et al [69], 2021	US	100	Encoder-decoder	DSC^f^: 0.7423	Introduces edge operators
	Mi et al [70], 2021	US	430	UNet	DSC: 0.780	A 3-branch network design; uses prior knowledge of the carotid artery
	Latha et al [71], 2022	US	361	CapsuleNet	Accuracy: 0.967	Compares multiple DL^g^ and ML^h^ models
	Jiang et al [72], 2023	US	244	CNN^i^	DSC: mean 0.927, SD 0.054	Introduces the concept of centerline extraction
**AI-assisted detection of AF^j^ and LA^k^ enlargement**						
	Degel et al [73], 2018	US	161	CNN	DSC: mean 0.75, SD 0.17; HD^l^: mean 4.46, SD 2.73 mm	Introduces shape priors; introduces adversarial learning
	Moradi et al [74], 2019	US	1137	UNet	DSC: mean 0.945, SD 0.12; HD: mean 1.62, SD 0.05 mm	Combines a pyramid network with UNet; uses the Niblack global thresholding method
	Leclerc et al [75], 2020	US	2000	UNet	HD: mean 5.9, SD 3.6 mm	Cascaded network; presents a novel attention mechanism
	Liu et al [76], 2021	US	3500	UNet	DSC: 0.949; HD: 4.33 mm	Proposes a pyramid local attention module; proposes a label coherence learning mechanism
	Li et al [77], 2023	US	1764	ResNet^m^	DSC: mean 0.959, SD 0.002; HD: mean 0.422, SD 0.314 mm	Introduces residual and branched networks; introduces dilated convolutions; incorporates multiscale concepts in the decoder
**AI-assisted detection of WMH^n^**						
	Liu et al [78], 2020	MRI^o^	75	CNN	DSC: 93.49	Cascaded network; uses dense blocks and dilated blocks; uses multiscale processing
	Park et al [79], 2021	MRI	170	UNet	DSC: mean 0.8107, SD 0.0203	Incorporates an auxiliary classifier; applies the highlight foreground method; uses multimodal MRI images
	Li et al [80], 2021	MRI	40	UNet	DSC: mean 0.736, SD 0.074	Introduces squeeze-and-excitation blocks and dense connection
	Huang et al [81], 2023	MRI	455	VNet	DSC: mean 0.81, SD 0.06	Proposes the LSLoss^p^ loss function
	Farkhani et al [82], 2024	MRI	430	UNet	DSC: 0.8559	Incorporates consecutive self-attention encoders

^a^AI: artificial intelligence.

^b^CAS: carotid artery stenosis.

^c^US: ultrasound.

^d^RBF: radial basis function.

^e^SVM: support vector machine.

^f^DSC: Dice similarity coefficient.

^g^DL: deep learning.

^h^ML: machine learning.

^i^CNN: convolutional neural network.

^j^AF: atrial fibrillation.

^k^LA: left atrium.

^l^HD: Hausdorff distance.

^m^ResNet: residual network.

^n^WMH: white matter hyperintensities.

^o^MRI: magnetic resonance imaging.

^p^LSLoss: level set loss.

#### Risk Factors of Stroke

**Figure 6:**
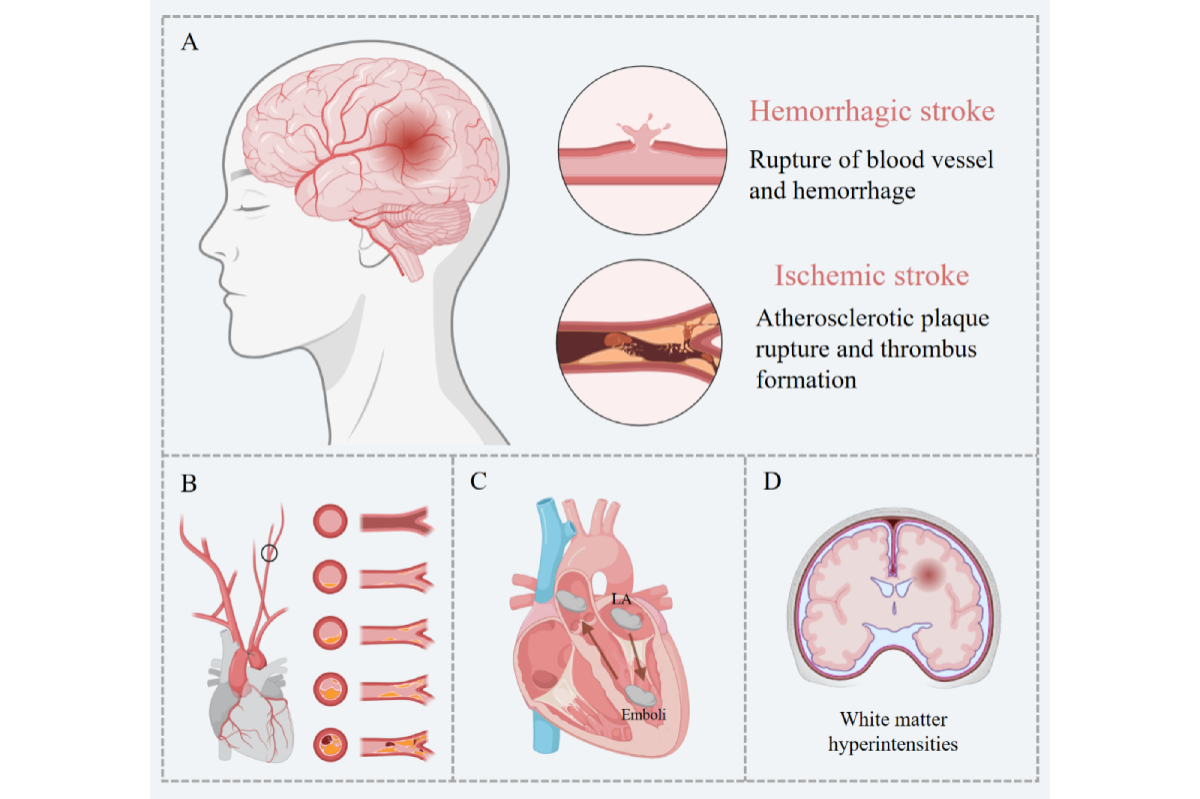
Risk factors and etiology of stroke. (A) Hemorrhagic stroke and ischemic stroke. (B) Large artery atherosclerosis–type stroke and its risk factors. (C) Cardioembolism-type stroke and its risk factors. (D) Small artery occlusion–type stroke and its risk factors. LA: left atrium.

Stroke can be divided into 3 distinct subtypes with well-defined etiologies: large artery atherosclerosis, cardioembolism, and small artery occlusion (Figure 6) [66].

Large artery atherosclerosis-type stroke is typically caused by atherosclerotic stenosis in the carotid and intracranial large arteries. For patients with mild stenosis, conservative treatment is recommended. For those with severe stenosis, carotid endarterectomy surgery is performed to prevent the occurrence of ischemic stroke [71].

Cardioembolism-type stroke is caused by cardiogenic diseases, where emboli from the heart and aortic arch dislodge and circulate, leading to cerebral artery embolism [83]. Studies have shown that left atrium (LA) enlargement may lead to myocardial fibrosis and disruption of atrial muscle bundles, resulting in blood stasis within the atrium and reduced atrial contraction capacity. This can lead to AF, thereby increasing the risk of cardioembolic stroke [84].

Small artery occlusion-type stroke, also known as lacunar stroke, is a pathological condition caused by disease in the small penetrating arteries of the deep regions of the cerebral hemispheres or brainstem, leading to ischemic necrosis of the brain tissue supplied by these arteries [85]. The international Standards for Reporting Vascular Changes on Neuroimaging-1 clearly state that white matter hyperintensities (WMH) are neuroimaging indicators of small vessel disease and can predict the occurrence of stroke [16].

#### AI-Assisted Detection of CAS

**Figure 7:**
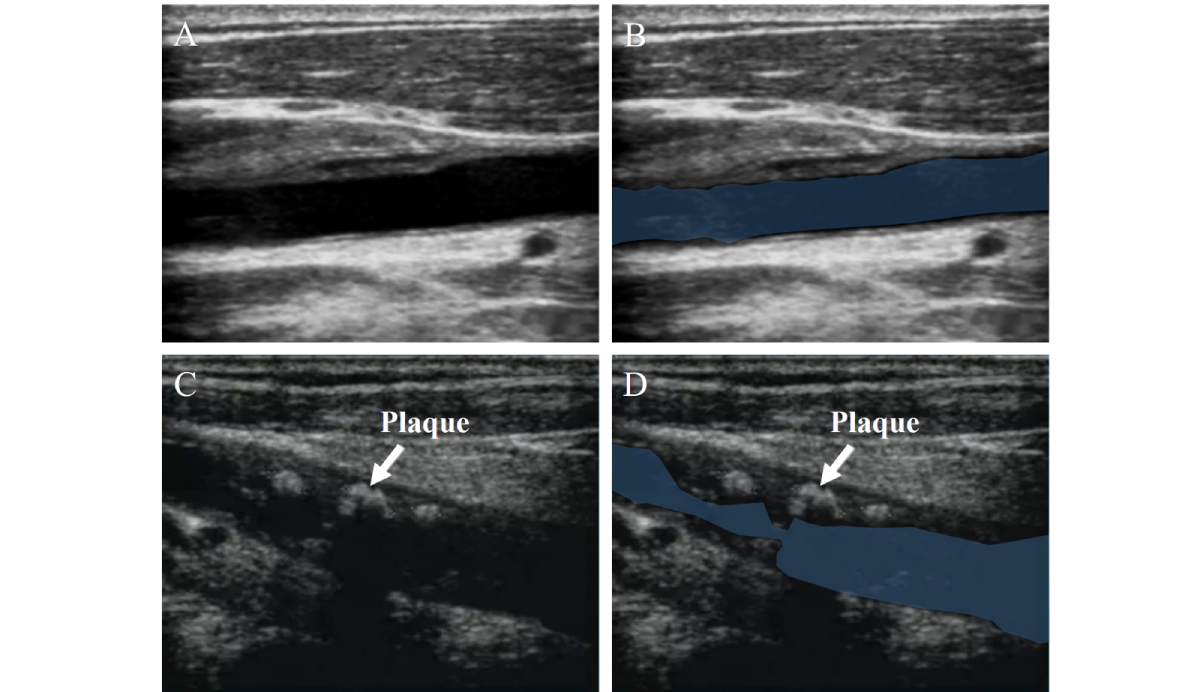
Carotid artery stenosis (CAS). (A) Healthy person. (B) Healthy person (annotated). (C) Patient with CAS. (D) Patient with CAS (annotated). Lumens are marked in blue.

The most commonly used imaging method for the detection of CAS in clinical practice is ultrasound (Figure 7) [86,87].

In 2015, Alam et al [67] proposed a robust fuzzy radial basis function network that combines fuzzy c-means clustering and a radial basis function network, incorporating spatial information and a smoothing parameter to handle noise.

In 2017, Araki et al [68] combined improved spatial domain filtering techniques with ML to use the morphological differences in carotid artery walls from ultrasound images. The authors used the improved spatial domain filtering techniques to remove image noise and used SVMs for classification [68].

In 2021, Al-Mohannadi et al [69] introduced morphological operations in an encoder-decoder structure to reduce the impact of noise on the results, using Sobel gradient direction images and Prewitt gradient direction images as inputs. In the same year, Mi et al [70] designed an MBFF-Net (a multibranch feature fusion network) with 3 branches. The first 2 branches extract plaque features at multiple scales and different contexts, while the third branch uses prior information [70].

In 2022, Latha et al [88] compared various ML and DL algorithms for carotid artery segmentation. Among the ML algorithms, RF performed the best, while CapsuleNet outperformed other DL algorithms [88].

In 2023, Jiang and Chiu [72] integrated a centerline extraction network and a dual-stream centerline-guided network into a 3D UNet. The centerline extraction network generates centerline heat maps to indicate the position of the carotid artery centerline, while the centerline-guided network segments the 3D ultrasound images based on the centerline heat maps [72].

#### AI-Assisted Detection of AF and LA Enlargement

**Figure 8:**
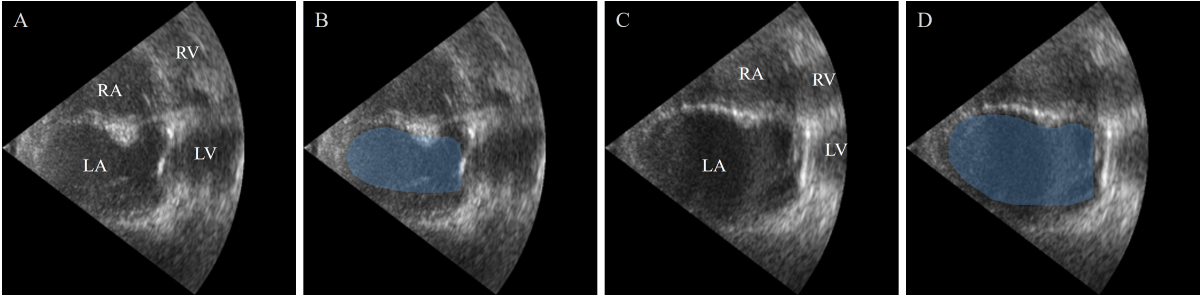
The presence of left atrium (LA) enlargement. (A) Healthy person. (B) Healthy person (annotated). (C) Patient with LA enlargement. (D) Patient with LA enlargement (annotated). LA areas are marked in blue. LV: left ventricle; RA: right atrium; RV: right ventricle.

Similar to CAS, echocardiography is commonly used clinically to detect stroke-related heart disease, such as AF and LA enlargement (Figure 8) [15].

In 2018, Degel et al [73] proposed an algorithm that combines DL, shape priors, and adversarial learning to achieve automatic segmentation of the LA in 3D ultrasound images. This algorithm uses VNet for 3D volume segmentation and trains an autoencoder network to constrain the segmentation results to the shape of the LA [73].

In 2019, Moradi et al [74] proposed MFP-UNet, which combines a pyramid network with the UNet model, adding 2 downsampling layers to extract more detailed features from the images. The authors also used the Niblack global thresholding method to preprocess the cardiac ultrasound images to enhance contrast [74].

In 2020, Leclerc et al [75] proposed an RUNet composed of 2 UNets. The first UNet obtains the region of interest by dilating the preliminary results, and the second UNet is used to predict the final segmentation. RUNet outperformed other networks in reducing geometric and anatomical prediction anomalies [75].

In 2021, Liu et al [76] introduced a pyramid-shaped local attention mechanism in UNet to capture contextual information and designed a label consistency learning mechanism to improve the classification accuracy of edge pixels in cardiac ultrasound images.

In 2023, Li et al [77] proposed a multitask DL model for cardiac ultrasound segmentation and key point detection, named EchoEFNet. The network introduces ResNets, branched networks, and dilated convolutions in the encoder part and incorporates multiscale concepts in the decoder part, combining both low-level and high-level features [77].

#### AI-Assisted Detection of WMH

**Figure 9:**
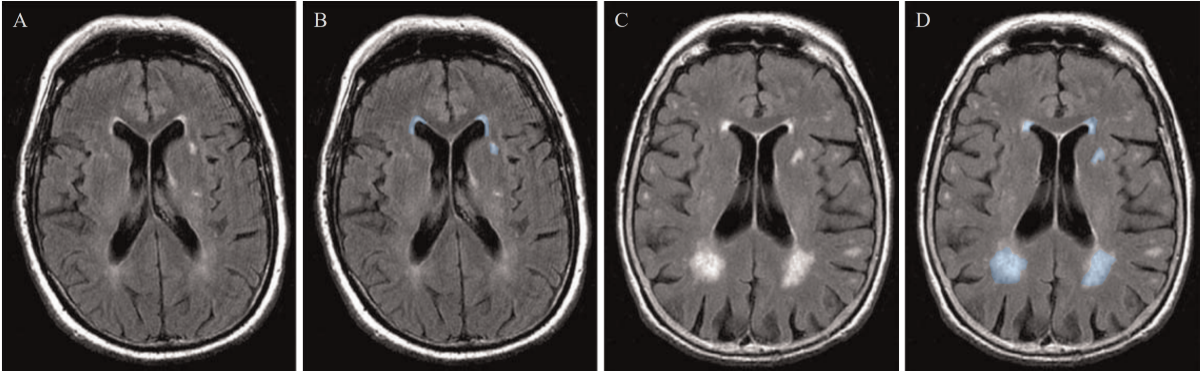
The presence of white matter hyperintensities (WMH). (A) Patient with minor WMH. (B) Patient with minor WMH (annotated). (C) Patient with extensive WMH. (D) Patient with extensive WMH (annotated). WMH areas are marked in blue.

MRI is superior to CT in terms of soft tissue contrast, allowing for better differentiation between white matter and gray matter in the brain. Therefore, MRI is more commonly used clinically to detect cerebral small vessel disease and WMH (Figure 9) [89,90].

In 2020, Liu et al [78] proposed the M2DCNN, a network composed of 2 symmetrical U-shaped subnetworks. This network uses dense blocks, dilated blocks, and multiscale processing to improve feature extraction and prediction capabilities. In addition, it introduces a loss function based on the similarity between lesions and background [78].

In 2021, Park et al [79] proposed a UNet with multiscale foreground highlighting, incorporating an auxiliary classifier in the middle layers of the decoder for deep supervision training. The authors used multiscale label images and applied the highlight foreground method to enhance foreground voxels [79].

In 2021, Li et al [80] introduced squeeze-and-excitation blocks and dense connections into the UNet architecture to better capture spatial and multiscale semantic information.

In 2023, Huang et al [81] proposed the level set loss (LSLoss) loss function. LSLoss consists of 4 loss terms, each designed to optimize foreground loss, background loss, region of interest loss, and divergence loss, to improve the accuracy of segmentation results [81].

In 2024, Farkhani et al [82] proposed the volumetric segmentation of WMH using transformer architecture, which enhances the UNet by using consecutive self-attention encoders to capture spatial dependencies in the data.

#### In-Depth Analysis of Stroke Risk Prediction

Carotid and cardiac ultrasound segmentation can assist physicians in quantitatively assessing the condition of a patient’s heart and carotid arteries, providing important references for evaluating the risk of stroke. However, ultrasound image segmentation is a complex task. Compared to CT and MRI images, ultrasound images have lower contrast, noisy and blurry boundaries, and numerous artifacts, making ultrasound medical image segmentation more challenging.

For ultrasound image segmentation and classification, DL generally outperforms ML. Among ML algorithms, RF, with its capability to handle multifeature data, performs the best in handling these tasks with high accuracy and robustness. By contrast, other ML algorithms such as SVM and kNN perform well in certain specific tasks but are generally not as stable and accurate as RF. This is similar to the excellent performance of RF in stroke lesion segmentation and classification as discussed in the Segmentation and Classification of Stroke Lesions subsection, further demonstrating RF’s superior performance in image segmentation and classification tasks [88].

To address the significant noise in ultrasound images, many researchers have incorporated denoising techniques into their algorithms. Alam et al [67] introduced spatial information and smoothing parameters to handle noise, and Araki et al [68] used spatial filtering techniques. In addition, due to the high noise and blurry boundaries in ultrasound images, advanced architectures such as transformers may not necessarily outperform traditional convolutional blocks. In the comparative experiments in the study by Liu et al [76], traditional architectures such as UNet, UNet++, and ResNet performed better than Swin-UNet. Although Swin-UNet, with its advanced transformer architecture, significantly improved feature extraction and generalization capabilities, it exhibited overfitting in the segmentation of edge regions due to the substantial noise and blurred edges in ultrasound images.

The structures of carotid and cardiac ultrasound images are relatively fixed, and the introduction of morphological operations can better optimize these fixed structures. This effectively reduces the impact of noise and small artifacts, improves segmentation accuracy, enhances structural features, and increases robustness. Araki et al [68] used the morphological characteristics of the carotid artery wall to specifically improve spatial filtering, while Al-Mohannadi et al [69] introduced Sobel and Prewitt operators to make the model more attentive to edge information, aiding in the identification and segmentation of the boundaries of the carotid artery and the heart. Mi et al [70] and Degel et al [73] incorporated shape prior information, and Liu et al [76] introduced a label consistency learning mechanism. These 3 studies used the concept of templates to constrain the segmentation results and reduce missegmentation. For the segmentation of carotid vessels, centerline extraction can accurately determine the center position of the vessels, providing precise references for subsequent segmentation and feature extraction. This can largely ignore small noise and artifacts in the image, providing more stable segmentation results [72].

For WMH segmentation, many researchers have attempted to use attention mechanisms to enhance the model’s focus on small targets. The attention mechanism allows the model to selectively focus on important regions of the image based on the task requirements, reducing dependence on irrelevant information [78]. Farkhani et al [82] achieved the precise capture of key regions through self-attention and hybrid transformer mechanisms, contributing to the improvement of WMH segmentation accuracy.

In the task of WMH segmentation, there is a significant class imbalance due to the large difference in the number of pixels between white matter and non–white matter regions. The WMH segmentation model tends to predict the more numerous classes while ignoring the minority classes, leading to overfitting. To address this issue, Liu et al [78] and Li et al [80] introduced dense blocks, which reduce the number of training parameters and alleviate the problem of vanishing gradients.

For small target segmentation, optimizing the loss function is a promising approach. In the study by Huang et al [81], an LSLoss function was proposed to train the segmentation network. This method allows for training without actual ground truth labels, helping to avoid overfitting the training data.

### Stroke Prognosis

#### Overview

Stroke prognosis refers to predicting long-term outcomes such as recovery status, quality of life, and survival rates of patients based on medical imaging and clinical dates. AI technology can quantify the extent of brain tissue damage, assess patients’ prognostic situations, and provide benchmarks for long-term rehabilitation (Table 4) [91].

**Table 4:** Methods and performance of stroke prognosis.

Study, year		Image type	Data set, n	Methods	Performance	Merits
**Estimation of final infarct lesion and penumbra**						
	McKinley et al [92], 2017	MRI^a^	80	RF^b^	AUC^c^: mean 0.94, SD 0.08	An automated method to estimate the penumbra; uses multimodal MRI images
	Nielsen et al [93], 2018	MRI	222	CNN^d^	AUC: mean 0.88, SD 0.12	Integrates time to maximum; uses apparent diffusion coefficient threshold
	Yu et al [94], 2020	MRI	182	UNet	AUC: 0.92; DSC^e^: 0.58	Does not depend on reperfusion information
	Pinto et al [95], 2021	MRI	75	CNN	DSC: 0.38; HD^f^: 29.21 mm	Accounts for the underlying cerebral blood flow; combines RBM^g^ and GRU^h^
	Wong et al [96], 2022	MRI	100	UNet	DSC: mean 0.88, SD 0.01; accuracy: mean 0.75, SD 0.03; AUC: mean 0.80, SD 0.03	Incorporates rotation and reflection variants; incorporates clinical indicators; predicts the modified Rankin Scale outcomes
**Estimation of stroke recovery outcomes**						
	Bentley et al [97], 2014	CT^i^, CRs^j^	116	SVM^k^	AUC: 0.744	Multimodal CRs
	Monteiro et al [98], 2018	CRs	541	RF	AUC: mean 0.808, SD 0.085	Compares RF, XGBoost^l^, and logistic regression
	Cheon et al [99], 2019	CRs	15,099	DNN^m^	AUC: 0.8348	Incorporates PCA^n^ into the DNN
	Kuang et al [100], 2019	CT	257	RF	AUC: 0.89	Introduces the ASPECTS^o^ scale
	Scrutinio et al [101], 2020	CRs	1207	RF	AUC: 0.928; accuracy: 0.863	Implements the synthetic minority oversampling technique
	Brugnara et al [102], 2020	CT, CRs	246	ML^p^	AUC: 0.856; accuracy: 0.804	Integrates multimodal CRs; predicts the modified Rankin Scale outcomes

^a^MRI: magnetic resonance imaging.

^b^RF: random forest.

^c^AUC: area under the curve.

^d^CNN: convolutional neural network.

^e^DSC: Dice similarity coefficient.

^f^HD: Hausdorff distance.

^g^RBM: restricted Boltzmann machine.

^h^GRU: gated recurrent unit.

^i^CT: computed tomography.

^j^CR: clinical record.

^k^SVM: support vector machine.

^l^XGBoost: extreme gradient boosting.

^m^DNN: deep neural network.

^n^PCA: principal component analysis.

^o^ASPECTS: Alberta Stroke Program Early Computed Tomography Score.

^p^ML: machine learning.

#### Estimation of Final Infarct Lesions and Penumbra

Acute stroke lesions tend to increase in size over time, leading to increased damage to brain tissue. To address this issue, some researchers have used AI to predict the final infarct lesions and the penumbra [103].

In 2017, McKinley et al [92] proposed an RF-based algorithm for penumbra prediction in multimodal MRI, called FASTER. This algorithm first extracts various statistical metrics from perfusion and diffusion imaging in the MRI data and then uses a decision forest algorithm to predict the penumbra [92].

In 2018, Nielsen et al [93] used CNNdeep to predict final infarct lesion, outperforming traditional general linear models, CNNs based on time to maximum, and apparent diffusion coefficient threshold methods.

In 2020, Yu et al [94] proposed a UNet-based model for predicting the final infarct lesions in patients with acute ischemic stroke. This model is the first DL-based stroke infarct prediction model that does not rely on reperfusion information. The volume error between the predicted results and the ground truth is only 9 mm^3^ [94].

In 2021, Pinto et al [95] proposed a method combining restricted Boltzmann machine and gated recurrent unit to predict the final stroke lesion in patients with acute ischemic stroke after 90 days. Each restricted Boltzmann machine learns features from different MRI parameter maps, which are then merged with the original maps and input into a convolutional and recurrent neural network architecture for supervised learning [95].

In 2022, Wong et al [96] developed a DL-based method for predicting the final infarct volume from MRI images. This approach uses networks with rotation and reflection variants and incorporates clinical variables to predict the 90-day modified Rankin Scale score [96].

#### Estimation of Stroke Recovery Outcomes

Clinical data can assist in estimating stroke recovery. Lesion volume and lesion location are critical factors in predicting recovery. Patients with extensive brain damage or strokes affecting critical brain areas generally have a poorer prognosis. Integrating patient clinical records with medical imaging can enhance the estimation of recovery outcomes [104].

Bentley et al [97] used SVMs to predict whether patients with acute ischemic stroke would develop symptomatic intracerebral hemorrhage after thrombolysis. Through the analysis of clinical records and brain CT images of 116 patients with acute stroke, the authors found that SVMs outperformed traditional prediction scoring systems in predicting symptomatic intracerebral hemorrhage [97].

Monteiro et al [98] investigated the use of ML methods to predict functional recovery in patients with ischemic stroke 3 months after the event. The study examined the performance of 5 different classifiers, among which RF exhibited the best performance [98].

Cheon et al [99] constructed a deep neural network model combined with principal component analysis for feature extraction. The model demonstrated high predictive ability based on clinical records from 15,099 patients with stroke [99].

Kuang et al [100] proposed an automated method for Alberta Stroke Program Early CT Score scoring from NCCT images of patients with acute ischemic stroke. This method uses texture features to train an RF classifier and performs well in identifying large areas of early infarction [100].

Scrutinio et al [101] used an RF algorithm enhanced with synthetic minority oversampling technique to predict the 3-year mortality rate of patients with severe stroke, significantly outperforming the logistic regression model.

Brugnara et al [102] integrated clinical records, multimodal imaging, and angiographic features to predict the modified Rankin Scale outcomes of patients with acute ischemic stroke after endovascular treatment. With the increase in medical information modalities, the model performance gradually improves [102].

#### In-Depth Analysis of Stroke Prognosis

For stroke prognosis, both DL and ML have their respective advantages [105]. DL models require large amounts of data for training to learn sufficiently complex features and avoid overfitting. This also explains why the deep neural network in the study by Cheon et al [99] achieved better performance after being trained on 15,099 clinical records, although acquiring a large amount of high-quality clinical data for stroke prognosis may be challenging.

By contrast, traditional ML methods have demonstrated greater robustness and efficiency in handling stroke prognosis tasks. Studies have shown that when handling stroke prognosis tasks, RF and its variant models have shown better performance than other ML algorithms [98,100,101]. This is mainly due to the robustness of RF models against data noise.

For the estimation of the final infarct lesion and penumbra, although the existing estimation may not achieve high DSC values, metrics such as volume error and AUC have reached high levels, indicating good practical value. In the future, through continual optimization and improvement of algorithms, the clinical translation prospects of AI in final infarct estimation will be even broader.

## Discussion

### Principal Findings

Over the past 25 years, significant advances have been made in AI-assisted stroke diagnosis. From 1999 to 2012, development was constrained by the limited performance of traditional thresholding and heuristic algorithms, resulting in low diagnostic accuracy and restricted clinical applicability. From 2012 to 2016, the advent of, and the advances made by, ML algorithms significantly expanded research in AI-assisted stroke diagnosis. The implementation of advanced statistical models and classifiers enabled more detailed analysis of stroke imaging data and improved diagnostic precision. After 2016, DL techniques, particularly CNNs, demonstrated significant improvements over traditional methods and ML algorithms [106].

AI has significantly enhanced the accuracy and efficiency of stroke lesion segmentation and classification, stroke risk prediction (eg, assessment of LA enlargement, CAS, and WMH), and stroke prognosis (eg, prediction of stroke recovery outcomes, final infarct lesion, and penumbra). Stroke lesion segmentation and classification enable more precise diagnoses, reduce the time to diagnosis, and improve patient treatment outcomes. Stroke risk prediction allows for timely intervention, potentially reducing stroke occurrence. Stroke prognosis helps physicians develop personalized rehabilitation plans, optimize treatment strategies in real time, and provide more accurate prognostic information to patients and their families. Integrating AI into clinical practice enables health care providers to use advanced data analysis and pattern recognition capabilities to streamline workflows, reduce diagnostic errors, enhance the diagnostic process, and improve the continuous monitoring and management of patients with stroke [107].

However, notwithstanding the aforementioned advances, several challenges remain in clinical applications. First, stroke diagnosis is a complex process that requires the integration of multimodal information (eg, medical imaging, genomic data, and clinical records) for comprehensive evaluation. However, the effective integration of multimodal data with diverse formats is challenging. Second, AI algorithms lack effective clinical validation. The generalization ability of AI models is limited, with many algorithms performing inconsistently across different data sets and imaging devices. In addition, given the inherent “black box” nature of AI algorithms, it is essential to develop transparent and interpretable models to help clinicians build trust in AI-assisted diagnosis. Finally, the use of AI in stroke diagnosis presents privacy and data security issues. Traditional data-sharing and model training methods may lead to information leakage.

The recent emergence of models such as the Segment Anything Model (SAM) [108], Vision Mamba (VMamba) [109], and multimodal large language models (LLMs) [110] has provided new directions for stroke diagnosis. SAM boasts a large parameter count and introduces pretrained weights from vision transformer, resulting in excellent generalization performance for medical image segmentation [108]. MedSAM, a fine-tuned version of the original SAM tailored for medical images, shows a 22.51% improvement in DSC on zero-shot medical image segmentation tasks compared to the untuned SAM [111]. nnSAM integrates pretrained SAM encoders and UNet encoders in parallel, combining robust feature extraction capabilities with better adaptability to different image segmentation tasks [112]. SAM performs well on external data sets from various imaging devices without further training, demonstrating potential to address the issue of limited generalization ability.

Multimodal LLMs can effectively integrate multimodal clinical data, enabling a more comprehensive understanding of patient conditions [113,114]. Gu et al [115] used LLMs for stroke quantitative evaluation using multimodal data, achieving an interrater agreement of 0.823 with expert ratings, demonstrating outstanding performance and application prospects. Meanwhile, VMamba represents a further improvement and upgrade of the transformer architecture. It can capture global information while maintaining linear computational complexity, addressing the performance bottleneck caused by the quadratic computational complexity of transformers [109]. Whether VMamba can achieve a leap in performance for stroke diagnosis similar to transformers remains to be seen and will require further evaluation over time.

Federated learning is able to address data privacy and security issues, allowing different institutions to collaboratively train AI models without sharing raw data. Federated learning not only protects patient privacy but also facilitates cross-institutional data sharing and collaboration, improving the generalization ability and accuracy of the models [116].

Overall, standardization is a key factor in promoting the application of AI in the medical field. It is necessary to establish unified evaluation standards and operational procedures to ensure that different institutions and researchers can consistently use AI technologies. In addition, medical professionals need continuous education and training on AI to better understand and trust the technology, which will enable them to apply it more effectively in clinical practice [117].

### Conclusions

This paper reviewed the current status, challenges, and development trends of AI in acute stroke lesion segmentation and classification, stroke risk prediction, and stroke prognosis over the past 25 years. AI-assisted stroke diagnosis has now shown good performance, assisting physicians in making rapid diagnoses and improving patient outcomes. With the development of new AI technologies such as SAM, LLMs, and VMamba, AI-assisted diagnosis of acute stroke is expected to achieve higher accuracy and stability in the future.

## Appendix

Multimedia Appendix 1 PRISMA_2020_checklist.

## Abbreviations

AF: atrial fibrillation
AI: artificial intelligence
AUC: area under the curve
CAS: carotid artery stenosis
CNN: convolutional neural network
CT: computed tomography
CTA: computed tomography angiography
CTP: computed tomography perfusion
DL: deep learning
DSC: Dice similarity coefficient
DWI: diffusion-weighted imaging
FLAIR: fluid-attenuated inversion recovery
kNN: k-nearest neighbors
LA: left atrium
LLM: large language model
LSLoss: level set loss
ML: machine learning
MRI: magnetic resonance imaging
NCCT: noncontrast computed tomography
PRISMA: Preferred Reporting Items for Systematic Reviews and Meta-Analyses
ResNet: residual network
RF: random forest
SAM: Segment Anything Model
SVM: support vector machine
T1WI: T1-weighted imaging
T2WI: T2-weighted imaging
VMamba: Vision Mamba
WMH: white matter hyperintensities
